# The clinical significance of mitochondrial calcium uniporter in gastric cancer patients and its preliminary exploration of the impact on mitochondrial function and metabolism

**DOI:** 10.3389/fonc.2024.1355559

**Published:** 2024-04-26

**Authors:** Zipeng Xu, Xia Chen, Haicun Zhou, Luming Sun, Ruobing Bai, Wenwen Yu, Junhao Yang, Hongbin Liu

**Affiliations:** ^1^ Lanzhou University Second Hospital, Lanzhou University, Lanzhou, China; ^2^ Department of General Surgery, Chang An Hospital, Xian, China; ^3^ Gansu Provincial Key Laboratory of Stem Cell and Gene Medicine, The 940th Hospital of Joint Lohistica Support force of Chinese People’s Liberation Army, Lanzhou, China

**Keywords:** gastric cancer, mitochondrial calcium uniporter, mitochondrial, metabolism, biosynthesis

## Abstract

**Objective:**

The objective of this study is to elucidate the influence of MCU on the clinical pathological features of GC patients, to investigate the function and mechanism of the mitochondrial calcium uptake transporter MCU in the initiation and progression of GC, and to explore its impact on the metabolic pathways and biosynthesis of mitochondria. The ultimate goal is to identify novel targets and strategies for the clinical management of GC patients.

**Methods:**

Tumor and adjacent tissue specimens were obtained from 205 patients with gastric cancer, and immunohistochemical tests were performed to assess the expression of MCU and its correlation with clinical pathological characteristics and prognosis. Data from TCGA, GTEx and GEO databases were retrieved for gastric cancer patients, and bioinformatics analysis was utilized to investigate the association between MCU expression and clinical pathological features. Furthermore, we conducted an in-depth analysis of the role of MCU in GC patients. We investigated the correlation between MCU expression in GC and its impact on mitochondrial function, metabolism, biosynthesis, and immune cells. Additionally, we studied the proteins or molecules that interact with MCU

**Results:**

Our research revealed high expression of MCU in the GC tissues. This high expression was associated with poorer T and N staging, and indicated a worse disease-free survival period. MCU expression was positively correlated with mitochondrial function, mitochondrial metabolism, nucleotide, amino acid, and fatty acid synthesis metabolism, and negatively correlated with nicotinate and nicotinamide metabolism. Furthermore, the MCU also regulates the function of the mitochondrial oxidative respiratory chain. The MCU influences the immune cells of GC patients and regulates ROS generation, cell proliferation, apoptosis, and resistance to platinum-based drugs in gastric cancer cells.

**Conclusion:**

High expression of MCU in GC indicates poorer clinical outcomes. The expression of the MCU are affected through impacts the function of mitochondria, energy metabolism, and cellular biosynthesis in gastric cancer cells, thereby influencing the growth and metastasis of gastric cancer cells. Therefore, the mitochondrial changes regulated by MCU could be a new focus for research and treatment of GC.

## 1 Introduction

In the global context of malignant tumors, gastric cancer (GC) ranks fourth in incidence and third in mortality. Current treatment methods for gastric cancer primarily involve surgical resection, as well as options such as radiotherapy, chemotherapy, targeted therapy, and immunotherapy. Despite the variety of treatment methods, the prognosis for patients with GC remains poor (1), with a low five-year survival rate (2). Therefore, it is particularly urgent and important to understand the mechanisms of GC occurrence and development, refine treatment methods for GC, and improve the survival status of GC patients.

Mitochondria are organelles found within eukaryotic cells that play a role in cellular energy metabolism and homeostasis. One of the common characteristics of cancer cells is the presence of severe mitochondrial dysfunction, indicating the significant role of mitochondrial dysfunction in the development of cancer. Mitochondrial calcium uptake is crucial for regulating cellular signaling events, energy status, production of reactive oxygen species (ROS), and cell survival (3). Ca^2+^ is the most abundant second messenger in human cells and plays various roles in fundamental cellular physiology, including regulating gene expression, controlling the cell cycle and proliferation, facilitating cell motility, regulating autophagy, and inducing apoptosis. Tumorigenic pathways are linked to changes or abnormal activation of Ca^2+^ channels or transporters.

Intracellular Ca^2+^ homeostasis is primarily regulated by mitochondria, and the uptake of mitochondrial Ca^2+^ depends on the mitochondrial calcium uniporter (MCU) (4, 5). Dysregulation of the MCU leads to abnormal uptake of Ca^2+^ by the mitochondria, resulting in an imbalance in Ca^2+^ homeostasis. Aberrant calcium (Ca^2+^) signaling is linked to cancer cell proliferation, adhesion, migration, invasion, and epithelial-mesenchymal transition (EMT) (6). Previous studies have indicated that the MCU complex and its regulatory proteins are often dysregulated in various cancers, such as breast cancer (BC), prostate cancer, ovarian cancer, and colorectal cancer (7–9). Moreover, aberrant expression of these MCU contributes to the proliferation, migration, invasion, and resistance to apoptosis of cancer cells, often associated with a poor prognosis in cancer patients (10).

From a therapeutic standpoint, mitochondrial metabolism therapy is an appealing approach for cancer treatment. The precise role of mitochondrial calcium uptake in the biology of GC has yet to be elucidated. Therefore, this study aims to comprehensively understand the role and mechanism of mitochondrial calcium uptake in the occurrence and development of GC, to elucidate the metabolic mechanisms of mitochondria and their biological signaling effects, and to clarify the impact of the MCU on the clinical pathological characteristics of GC patients and its effects on metabolism and biosynthesis. This research aims to identify new targets and approaches for the clinical diagnosis and treatment of GC.

## 2 Materials and methods

### 2.1 Tissue specimens

The tissue specimens were collected from patients who underwent radical gastrectomy for GC between 2018 and 2020. The specimens included GC tissue and paracancerous tissue. After the tissues were removed during surgery, they were immersed in tissue fixative (All specimens were fixed in the fixative and then preserved in paraffin, making it impossible to extract RNA and protein). All patients underwent preoperative gastrointestinal imaging, including MRI, CT, or gastroscopy, and the diagnosis of GC was confirmed by pathological biopsy. None of the patients had received antitumor therapy, including radiotherapy and chemotherapy, before surgery, and they had no serious chronic underlying diseases. Patients who died during the perioperative period, had concurrent malignant tumors, had tumor invasion of other organs or distant metastases, or did not achieve R0 resection were excluded. This study complied with the requirements of the Helsinki Declaration and was approved by the hospital’s medical ethics committee (2018KYLL001).

### 2.2 Collection of clinical data and follow-up

Collect clinical and laboratory examination data for all patients, including gender, age, height, weight, tumor differentiation, Lauren classification, maximum tumor diameter, metastatic lymph nodes, CEA, CA-199, etc. Follow-up will be conducted through outpatient visits and phone calls, with patients being monitored for 3 years after discharge to determine their survival status.

### 2.3 Data source and processing

Download RNA-seq data of GC patients and corresponding clinical information, including data from TCGA-STAD (32 cases of adjacent non-cancerous tissue and 375 cases of GC tissue), normal tissue data from the GTEx database (174 cases), and the GSE63089 dataset from the GEO database (45 pairs of GC and normal tissue). Standardize the data by filtering and applying log2 transformation to ensure consistency in data processing, while eliminating batch effects.

### 2.4 Data processing and analysis

The primary bioinformatics analysis software used in this study is R software (version 4.0.3), while the image processing software includes Microsoft PowerPoint, Adobe Photoshop, NDP view 2, Phen chart, Image Lab, and Adobe Illustrator CC 2019. RNA sequencing (RNA-seq) data and corresponding clinical and survival prognosis data are extracted from the aforementioned databases. The median value of MCU mRNA expression is used as the cutoff (>50% vs ≤50%) to divide the samples into high and low expression groups. The combined analysis of TCGA and GTEX (using FPKM data from USUC Xena, with logFC 1.0 and P-value 0.05) and single-gene analysis of MCU were performed to examine the expression, clinical pathological characteristics, and survival prognosis of MCU in GC. Additionally, GO analysis, KEGG analysis, protein-protein interaction network (PPI) network analysis, pathway enrichment analysis, and other related analyses are performed to study the molecular functions, biological pathways, cellular components, as well as the metabolism and expression levels of related molecules in relation to the MCU. Furthermore, the functional analysis of the MCU gene and alterations in associated molecular signaling pathways are also examined.

### 2.5 Immunohistochemistry

Tissues are immersed in a 4% paraformaldehyde fixative for more than 24 hours. After fixation, the tissues are sequentially immersed in 70% ethanol, 80% ethanol, 95% ethanol, 100% ethanol, xylene, and paraffin solution. After dehydration, clearing, and paraffin embedding, the tissues are placed in paraffin and allowed to cool and solidify before being sectioned. Tissue blocks are cut into 4-μm-thick sections and then attached to glass slides by baking. Subsequently, a blocking solution is added to the sections and left to incubate at room temperature for 30 minutes. After blocking, the primary antibody (MCU antibody, 1:500 dilution, ab272488, Abcam) is applied and incubated overnight. The following day, the secondary antibody is added and incubated at room temperature for 30 minutes. This is followed by sequentially adding DAB staining solution, hematoxylin staining solution, post-staining dehydration and clearing, and mounting with neutral gum. The tissue sections stained with immunohistochemistry are independently evaluated for H-SCORE by AI and two researchers. The H-score is calculated using the formula H-SCORE=∑(pi×i), where pi represents the percentage of positive cells among all cells in the section, and i represents the staining intensity.

### 2.6 Statistical analysis

Analysis is conducted using GraphPad Prism 8.0 and SPSS 22.0 statistical software. Normally distributed quantitative data is typically presented as Mean ± SD, and intergroup comparisons are usually conducted using t-tests. Count data is presented as absolute numbers or percentages, and intergroup comparisons are conducted using the χ^2^ test. Survival rates are determined using the Kaplan-Meier method, and survival analysis is conducted using the Log-rank test. A significance level of P< 0.05 is considered statistically significant.

## 3 Results

### 3.1 Clinical data

A total of 205 clinical records of patients who underwent radical surgery for GC were collected. The records included 153 males and 52 females, with an average age of (57.79 ± 11.72) years. Immunohistochemical staining was utilized to detect the expression of the MCU protein in the tumor tissues of 205 patients with gastric cancer. The scoring results revealed that the highest H-SCORE for MCU expression in tumor tissues of the 205 GC patients was 72.50, the lowest was 13.77, and the average score was 45.81 ± 14.49. Based on the mean value of 45.81, the patients were divided into a high-expression group (H-SCORE ≥ 45.81) and a low-expression group (H-SCORE< 45.81). There were no statistically significant differences (P > 0.05) in general data such as gender, age, BMI, tumor location, tumor stage, differentiation degree, and Lauren classification between the two groups, indicating comparability (see **Table 1**).

**Table 1:** Comparison of clinical data.

	Low(n=95)	High(n=110)	t/χ^2^	P
Gender				
female	25	27	0.084	0.771
male	70	83		
**Age**	57.91 ± 10.94	57.68 ± 12.40	0.136	0.892
**BMI**	22.51 ± 3.63	21.99 ± 4.17	0.940	0.349
Grade				
G1	4	5	0.232	0.891
G2	35	37		
G3	56	68		
Lauren Classification				
intestinal1type	45	50	0.081	0.960
diffuse1type	37	44		
mixed1type	13	16		

BMI, Body Mass Index.

### 3.2 expression of MCU in GC tissues

**Figure 1:**
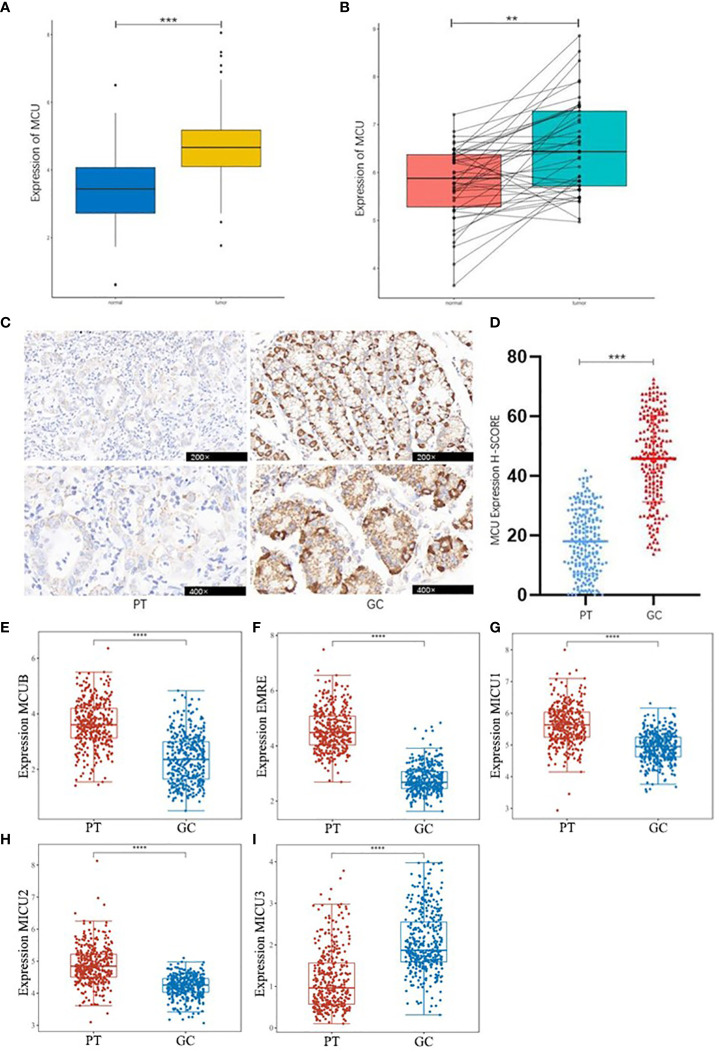
Differential Expression of MCU and MCU complex other subunits in GC. **(A)** Combined analysis of MCU expression in GC and normal gastric gland tissues from TCGA and GTEX databases. **(B)** Analysis of MCU expression in GC tissues and normal gastric gland tissues based on the GSE63089 dataset. **(C)** Immunohistochemical staining images of MCU expression in cancer tissues and adjacent tissues from patients. **(D)** Immunohistochemical H-SCORE ratings. **(E)** Expression of MCUB in gastric cancer tissues and adjacent tissues. **(F)** Expression of EMRE in gastric cancer tissues and adjacent tissues. **(G)** Expression of MICU1 in gastric cancer tissues and adjacent tissues. **(H)** Expression of MICU2 in gastric cancer tissues and adjacent tissues. **(I)** Expression of MICU3 in gastric cancer tissues and adjacent tissues **p<0.01, ***p<0.001, ****p<0.0001.

Based on the combined analysis of MCU mRNA expression data from 32 adjacent non-cancerous tissues and 375 GC tissues in the TCGA database, as well as 174 normal tissues from the GTEX database, and the analysis of MCU mRNA expression in 45 pairs of GC and normal tissues from the GEO database, it was found that the mRNA expression level of MCU is significantly upregulated in GC tissues compared to normal gastric gland tissues (P< 0.05), as shown in **Figures 1A**, **B**.

Immunohistochemical staining results of cancer tissues and paracancerous tissue from 205 GC patients indicated that MCU is highly expressed in GC tissues compared to paracancerous tissue (P< 0.05), as shown in **Figures 1C** , **D**. This is consistent with the mRNA expression results of GC patients in the database.

Then, we analyzed the expression of other subunits of the MCU complex, including MCUB, EMRE, MICU1, MICU2, and MICU3 in gastric cancer and adjacent tissues, and showed that MCUB, which co-composed of Ca^2+^ channels with MCU, was highly expressed in gastric cancer tissues, and the positive regulatory subunit MICU1 and MICU2 were highly expressed in gastric cancer tissues, and the negative regulatory subunit MICU3 was under expressed in gastric cancer tissues (P<0.05), as shown in **Figures 1E–I**.

### 3.3 Association of MCU expression with clinical pathological features of GC patients

We divided GC patients into high and low expression groups based on the median value of MCU mRNA expression (>50% vs. ≤50%). **Table 2** presents the correlation between MCU expression levels and the clinical pathological characteristics of GC patients. We found that general clinical pathological parameters (such as age and gender) did not show any significant statistical differences between the high and low expression groups (P ≥ 0.05). However, the high-expression group exhibited later T and N stages compared to the low-expression group (P< 0.05).

**Table 2:** MCU expression and clinicopathological features in the dataset.

	Overall (n=219)	Low (n=110)	High (n=109)	χ^2^	P
Factor(gender)					
female	90	45	45	0.003	0.955
male	129	65	64		
Factor(age group)					
≤62	85	41	44	0.221	0.638
>62	134	69	65		
Factor (ajcc pathologic stage)					
I, II	121	63	50	2.850	0.091
III, IV	98	47	59		
Factor (ajcc pathologic T)					
T1, T2	67	46	31	4.298	0.038*
T3, T4	152	64	78		
Factor (ajcc pathologic N)					
N0, N1	137	74	56	5.735	0.017*
N2, N3	82	36	53		
Factor (ajcc pathologic M)					
M0	202	101	94	1.747	0.186
M1	17	9	15		
Factor (treatment or therapy)					
no	149	72	79	1.035	0.309
yes	70	38	31		
Factor (OS)					
alive	164	82	74	1.183	0.277
dead	55	28	35		

*p<0.05.

TNM, tumor, node, metastasis.

Clinical pathological data and laboratory test results from 205 patients who underwent radical surgery for GC were collected. The clinical pathological features of the two patient groups were compared based on immunohistochemical scores. The results revealed that patients in the high expression group of MCU had later T stage (T3, T4 vs. T1, T2, P< 0.05) and N stage (N2, N3 vs. N0, N1, P< 0.05), and a larger tumor diameter (P< 0.05). Please refer to **Table 3**.

**Table 3:** Comparison of pathological data of MCU expression in clinical patients.

	Low(n=95)	High(n=110)	t/χ^2^	P
**CA19-9**	261.11 ± 1375.80	306.26 ± 1530.15	-1.371	0.172
**CEA**	10.28 ± 33.79	21.54 ± 78.03	-0.221	0.826
**Maximum tumour dimension**	4.74 ± 2.16	5.47 ± 2.46	-2.255	0.024*
T				
T1, T2	31	22	4.243	0.039*
T3, T4	64	88		
N				
N0, N1	41	31	7.225	0.007**
N2, N3	54	79		

*p<0.05, **p<0.01

T, tumor; N node.

Combining the mRNA analysis results from the database with the immunohistochemical examination results of clinical GC tissues, we discovered that high expression of MCU in GC tissues often indicates a more advanced T and N stage in patients.

### 3.4 MCU expression and the survival of GC patients

**Figure 2:**
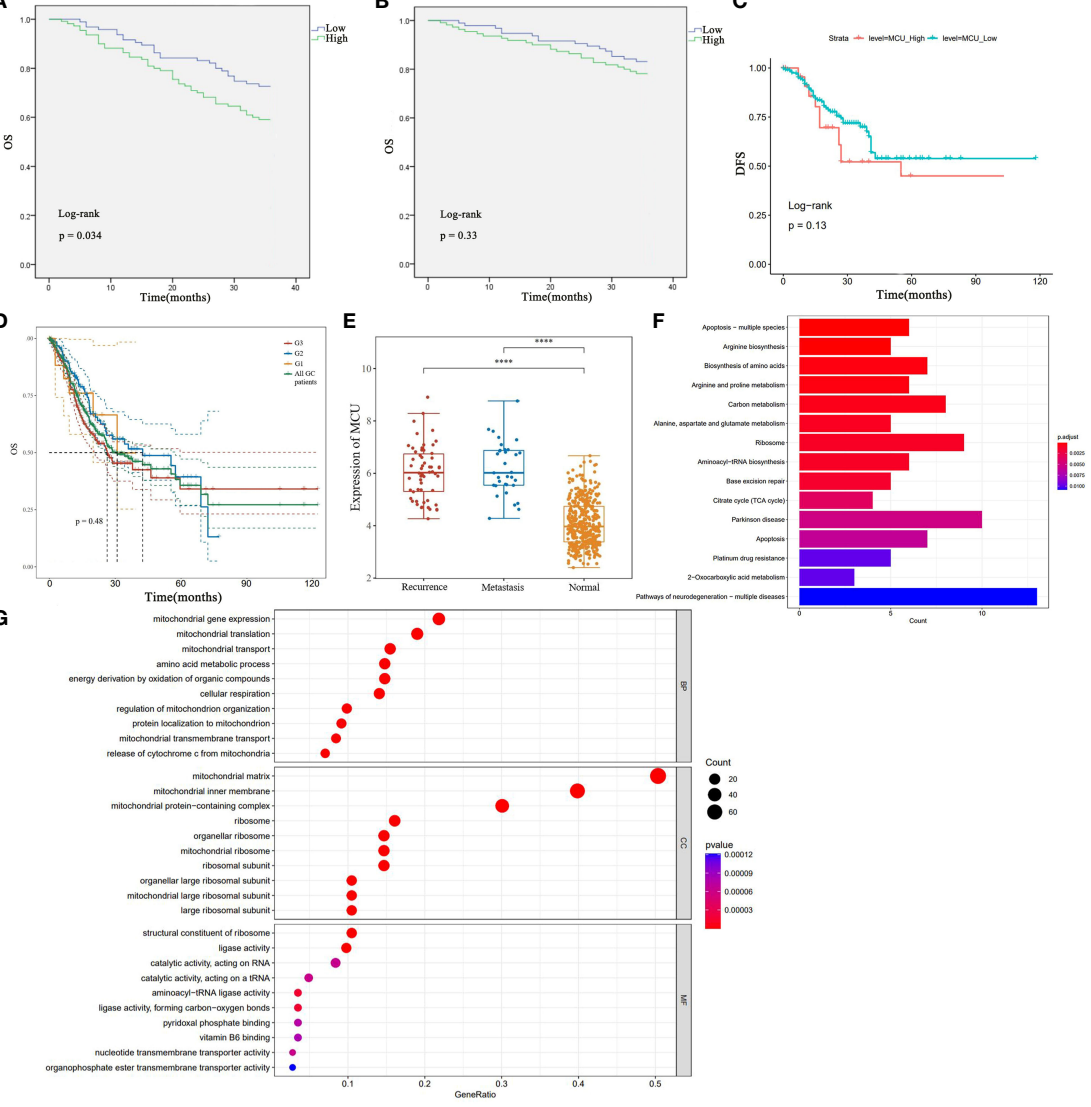
**(A)** Analysis of the Impact of High and Low MCU Expression on Overall Survival (OS) Based on Clinical GC Patient Data. **(B)** Analysis of the Impact of High and Low MCU Expression on Disease-Free Survival (DFS) in Clinical GC Patients. **(C)** Analysis of Overall Survival (OS) in High Expression Group and Low Expression Group in TCGA-STAD Cohort. **(D)** OS of patients with gastric cancer with different classifications (G1, G2, G3). **(E)** MCU expression in patients with recurrence and metastatic gastric cancer. **(F)** Results of KEGG Pathway Enrichment Analysis. **(G)** Results of GO Functional Enrichment Analysis. ****p<0.0001.

To elucidate the relationship between MCU expression and the prognosis of GC patients, we examined the influence of MCU expression on the survival outcomes of GC patients in both the TCGA cohort and clinical settings. Firstly, in the clinical patient cohort, we utilized the Kaplan-Meier method to compare the overall survival (OS) and disease-free survival (DFS) of patients with high MCU expression and low MCU expression. The study found that patients in the high MCU expression group had a shorter overall survival (3-year OS rate: 16.8% vs. 21.8%), but the difference was not statistically significant (P > 0.05), as shown in **Figure 2A**.

Further analysis of the disease-free survival of clinical patients revealed that the high mitochondrial calcium uniporter (MCU) expression group had a lower disease-free survival (3-year DFS), and the difference was statistically significant (P< 0.05), as shown in **Figure 2B**. Subsequently, in the TCGA-STAD cohort, we utilized the Kaplan-Meier method to compare the overall survival of the two groups of patients. The results indicated that patients in the high MCU expression group had a lower five-year survival rate (5-year OS), but the statistical difference was not significant (P > 0.05), as depicted in **Figure 2C**. After that, we studied the effect of MCU on the survival of GC patients in different categories (G1, G2, G3), and found that the expression of MCU (MCU expression was more than the Mean+SD of the normal group) in different categories had a similar effect on the survival of patients (5-year OS), see **Figure 2D**. The five-year survival rate of G1 patients with gastric cancer was (5-year survival rate, high group 48.28% vs low group 52.50%), the five-year survival rate of G2 patients with gastric cancer was (5-year survival rate, high group 41.45% vs low group 48.62%), and the five-year survival rate of G3 patients with gastric cancer was (5-year survival rate, high group 28.62% vs low group 33.45%). The survival rate of the high-expression MCU group was lower than that of the low-expression group in the gastric cancer patients with all three classifications. but the statistical difference was not significant (P > 0.05).

In order to understand the effect of MCU on recurrence and metastasis in gastric cancer patients, we detected the expression of MCU in patients with recurrence and metastatic gastric cancer after treatment, and found that MCU was highly expressed in the cancer tissues of patients with recurrent and metastatic gastric cancer, indicating that MCU may be associated with recurrence and metastasis of gastric cancer (P< 0.05), see **Figure 2E**.

### 3.5 The function and role of MCU in GC

In our previous studies, through previous studies we found that MCU is highly expressed in gastric cancer tissues, and that high expression of MCU is associated with worse tumor staging and prognosis. MCU acts as a channel for mitochondrial uptake of calcium ions, and how the high expression of MCU in gastric cancer has an impact on gastric cancer. To elucidate the impact of altered MCU expression on patients with GC, we initially performed KEGG pathway enrichment analysis to identify the potential signaling pathways associated with MCU. The results revealed that MCU is primarily enriched in apoptosis, biosynthesis of amino acids and metabolism, carbon metabolism, tricarboxylic acid (TCA) cycle, ribosome production and transport, and platinum drug resistance in GC (refer to **Figure 2F** for pathway details). Afterwards, we conducted GO functional enrichment analysis to investigate the potential roles of MCU, exploring the cellular components (CC), molecular functions (MF), and biological processes (BP) that may be affected by the altered expression of MCU. The results indicated that MCU primarily functions in mitochondria, influencing mitochondrial function, cytochrome C-related mitochondrial apoptosis, energy metabolism, and substance metabolism, including the biosynthesis of ribosomes, amino acids, nucleotides, and the transmembrane transport of substances (see **Figure 2G** for details).

To investigate the impact of MCU on mitochondrial function and metabolism in GC, we performed correlation analysis between the expression of MCU in GC patients and pathways or biomarkers associated with mitochondria and metabolism. The results revealed a positive correlation between MCU and factors associated with mitochondrial function and oxidative respiratory chain function, including MT-CO1, MT-CO2, MT-CO3, MT-CYB, and TFAM. Additionally, the expression of MCU showed positive correlations with cellular metabolic processes, such as DNA replication, the pentose phosphate pathway, pantothenate and CoA biosynthesis, glycerophospholipid metabolism, glycolysis, gluconeogenesis, and amino sugar and nucleotide sugar metabolism, while exhibiting a negative correlation with nicotinate and nicotinamide metabolism. Furthermore, the expression of MCU in GC patients was positively correlated with cell proliferation and ROS generation. (Refer to **Table 4** for details).

**Table 4:** MCU correlation analysis in gastric cancer patients.

	tStudent	p	$\hat{r}$ Pearson	CI95%	npairs
**Amino sugar and nucleotide sugar metabolism**	15.79	4.16e-04	0.18	0.08, 0.28	581
**ROS**	15.79	3.9e-04	0.18	0.08, 0.28	581
**Glycolysis Gluconeogenesis**	15.73	9.96e-0.6	0.23	0.12, 0.32	581
**Glycerophospholipid metabolism**	15.78	2.86e-04	0.19	0.08, 0.29	581
**Pantothenate and CoA biosynthesis**	15.84	0.009	0.13	0.03, 0.24	581
**Nicotinate and nicotinamide metabolism**	16.09	0.031	-0.11	-0.21, -0.01	581
**P53 pathway**	15.69	3.2e-07	0.26	0.16, 0.35	581
**Pentose phosphate pathway**	15.76	8.07e-05	0.20	0.10, 0.30	581
**Tumor proliferation signature**	15.85	0.012	0.13	0.03, 0.23	581
**DNA replication**	15.88	0.044	0.10	0.00, 0.21	581
**MT-CO1**	11.61	3.58e-28	0.43	0.37, 0.50	581
**TFAM**	11.16	2.52e-26	0.42	0.35, 0.49	581
**MT-CO2**	6.82	2.26e-11	0.27	0.20, 0.35	581
**MT-CO3**	9.44	9.11e-20	0.37	0.29, 0.43	581
**MT-CYB**	8.83	1.29e-17	0.34	0.27, 0.41	581

MT-CO1, mitochondrially encoded cytochrome c oxidase I; MT-CO2, mitochondrially encoded cytochrome c oxidase II; MT-CO3, mitochondrially encoded cytochrome c oxidase III; MT-CYB, mitochondrially encoded cytochrome b; TFAM, mitochondrial transcription factor A.

Therefore, our study has revealed that high expression of MCU in GC primarily promotes cancer progression by influencing mitochondrial function, including the tricarboxylic acid cycle, oxidative respiratory chain function, NAD^+^/NADH levels, and the regulation of DNA, amino acid, nucleotide, lipid, and energy substance synthesis and metabolism.

### 3.6 Correlation of MCU with immune cells

**Figure 3:**
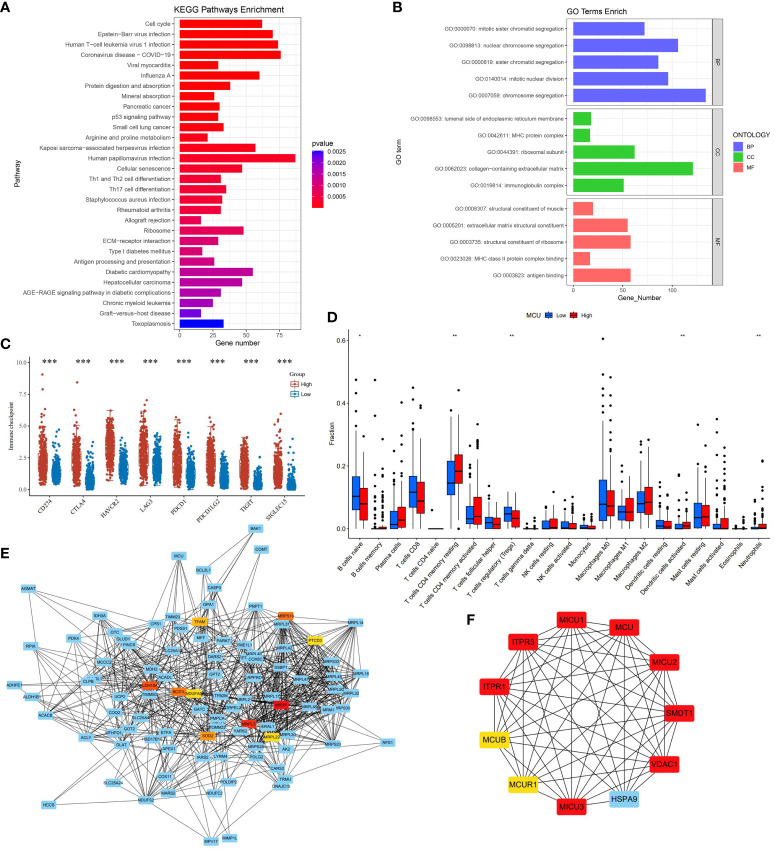
**(A)** The results of KEGG immune-related pathway enrichment analysis with MCU. **(B)** Results of MCU immune-related GO functional enrichment analysis. **(C)** Immune checkpoints associated with MCU expression in gastric cancer. **(D)** Correlation of MCU Expression with Immune Cells. **(E)** Protein-Protein Interaction Network (PPI) of MCU. **(F)** Top 10 Key Hub Genes Interacting with MCU. *p< 0.05, **p< 0.01, ***p<0.001.

In order to understand the effect of MCU on the tumor microenvironment and immune cells of gastric cancer patients, we first analyzed the pathways and mechanisms that MCU may affect immunity in gastric cancer patients, and the results showed that the effect of MCU expression on immunity was mainly enriched in MHC and MHC class II protein complex binding, immunoglobulin complex, antigen binding, Antigen processing and presentation, ECM−receptor interaction, Th1, Th2 and Th17 cell differentiation, Refer to **Figure 3A** for details. Then we analyzed the immune checkpoints again and obtained MCU-related immune checkpoints in gastric cancer patients, the results of which are shown in **Figure 3B**.

We analyzed the TCGA-STAD cohort data at the cellular level to investigate the relationship between MCU expression and immune cells in the tumor microenvironment. The results indicated that high expression of MCU is associated with a decrease in naive B cells and Tregs (T cells regulatory), and an increase in CD4^+^ memory resting T cells, activated dendritic cells, and neutrophils (P< 0.05). (Refer to **Figure 3C** for details).

### 3.7 Genes and proteins associated with MCU

To elucidate the mechanism of action of MCU in GC and understand the genes and proteins that interact with MCU, we constructed a PPI for MCU and analyzed the interacting genes and proteins. (Refer to **Figure 3D**). Furthermore, we conducted subnetwork selection for the entire PPI network and ranked the genes in the significant subnetwork based on their importance, ultimately identifying the top 10 key hub genes (Refer to **Figure 3E**). Among these, MICU1, MICU2, MICU3, MCUR1, MCUB, and SMDT1 are components of the MCU complex, collectively regulating the uptake of Ca^2+^ into the mitochondria. Inositol 1,4,5-trisphosphate receptor type 1 (ITPR1) and inositol 1,4,5-trisphosphate receptor type 3 (ITPR3) mediate intracellular Ca^2+^ release. VDAC1 is an anion channel protein situated on the outer membrane of mitochondria, while HSPA9 is mainly located within mitochondria and encodes a member of the heat shock protein 70 gene family.

## 4 Discussion

Gastric cancer (GC) is one of the leading causes of death globally. Despite the development of various treatment methods for GC, the five-year survival rate remains unsatisfactory. Therefore, it is crucial to identify new drug targets and develop effective treatments for GC. Based on research progress over the past few decades, energy and metabolic changes have been identified as one of the hallmarks of cancer. Recent studies have identified mitochondria as a crucial center that regulates cell fate by controlling bioenergetics and the production of various metabolites. Mitochondria, which are organelles within eukaryotic cells, typically number in the hundreds or thousands per cell and can occupy 25% of the cell cytoplasm. Mitochondria are involved in bioenergetic metabolism and cellular homeostasis, including oxidative phosphorylation (OXPHOS) for ATP generation, production of reactive ROS, as well as initiation and execution of apoptosis (3, 11). Mitochondrial dysfunction encompasses deficiencies in the tricarboxylic acid cycle enzymes, mutations in mitochondrial DNA genes, impairments in the mitochondrial electron transport chain, oxidative stress, and abnormal signaling of oncogenes and tumor suppressor genes. Severe mitochondrial functional abnormalities are frequently found in cancer cells. Mitochondrial dysfunction plays a critical role in tumorigenesis, impacting nearly all stages from tumor initiation to metastasis. Relevant studies have found that mitochondrial functional impairment increases the proliferation, migration, and chemoresistance of human GC cells (12, 13).

Calcium signaling is a crucial mechanism that rapidly translates signals in the tumor microenvironment into cellular responses. Ca^2+^ is an essential ion that regulates mitochondrial function. Mitochondrial calcium uptake is crucial for regulating mitochondrial signal transduction, energy status, ROS production, and cell apoptosis (3). Studies have shown that abnormal calcium (Ca^2+^) signaling is associated with the proliferation, adhesion, migration, invasion, and EMT of cancer cells (14). Mitochondrial Ca^2+^ uptake relies on the mitochondrial calcium uniporter (MCU), which is a channel responsible for mitochondrial Ca^2+^ uptake (4, 5). Current research has found that the expression of the MCU complex and its regulatory proteins is dysregulated in various types of cancer, such as breast cancer, ovarian cancer, and colorectal cancer (9, 15). Aberrant expression of the MCU promotes the proliferation, migration, invasion, and anti-apoptotic properties of cancer cells and is often associated with poor prognosis in cancer patients (10). In our study, we found that compared to healthy individuals and paracancerous tissu, GC patients’ cancer tissues exhibit high expression of MCU. Furthermore, patients with high MCU expression often have larger tumor volumes, worse T and N staging, and a poorer prognosis.

Why does the high expression of MCU lead to a poorer prognosis for patients with GC? Through data analysis, we found that MCU expression changes may widely affect mitochondrial function in gastric cancer patients, including energy production and a variety of bio anabolic processes including the synthesis and metabolism of amino acids, nucleotides, proteins, lipids, and NAD^+^/NADH. Rapidly dividing tumor cells require ample energy to support their proliferation, and mitochondria supply ATP to facilitate cell proliferation through OXPHOS. Glucose taken in by the body undergoes glycolysis to produce pyruvate. The pyruvate is then transported to the mitochondria and utilized to generate ATP through the TCA cycle and oxidative phosphorylation in aerobic conditions. Under hypoxic conditions within the cell, pyruvate is converted to lactate by the enzyme lactate dehydrogenase (LDH) and NADH, producing energy and NAD^+^. The dysregulation of the MCU leads to abnormal uptake of Ca^2+^ by the mitochondria, resulting in a disruption of Ca^2+^ homeostasis. The three principal enzymes of the TCA cycle (pyruvate dehydrogenase, isocitrate dehydrogenase, and α-ketoglutarate dehydrogenase) are all Ca^2+^-dependent dehydrogenases. Therefore, any disruption in Ca^2+^ homeostasis impacts the TCA cycle and its resulting products, such as ATP, NADPH, NADH, and others. Abnormal NADH generation also impacts lactate formation. Due to the Warburg Effect in cancer cells (aerobic glycolysis in cancer cells, where even in the presence of sufficient oxygen, many cancer cells consume high levels of glucose and secrete high levels of lactate (16)), the complex I of the oxidative respiratory chain (electron transfer chain) regulates the ratio of NAD^+^/NADH within the cell. This ratio is a necessary auxiliary factor that supports the biosynthetic reactions required for proliferation (17). Therefore, the high expression of MCU provides energy support for cancer cell growth and metabolism.

In addition to synthesizing ATP, mitochondria also play a crucial role in the biosynthesis of lipids, amino acids, and nucleotides. Metabolites produced in the TCA cycle are also used in the synthesis of these biomolecules. Compared to ATP generation, these biosynthetic processes are more susceptible to changes in mitochondrial function. Lipids, as crucial metabolic products, can enhance cell proliferation by influencing the formation of cell membranes. They also provide cellular energy and participate in intercellular signal transduction. Furthermore, research suggests that cancer progression is associated with both fatty acid (FA) synthesis and FA oxidation. Enzymes involved in fatty acid β-oxidation are upregulated in various cancers, and inhibiting them has been shown to inhibit cancer progression (18). Our research has found that the MCU is associated with fatty acid synthesis and metabolism processes, such as glycerophospholipid metabolism, glycolysis, gluconeogenesis, the pentose phosphate pathway, and pantothenate and CoA biosynthesis. Additionally, transcriptomic analysis of GC patient samples has indicated that the upregulation of nucleotide biosynthesis genes is one of the most common metabolic alterations in various cancer types (19, 20). In addition to protein synthesis, amino acid metabolism is a mechanism through which cancer cells fulfill their energy requirements. Amino acid metabolism is closely linked to the regulation of cell survival and apoptosis, and it plays a crucial role in controlling the fate of both normal and cancer cells. Our research has revealed that the MCU is associated with DNA replication and amino sugar and nucleotide sugar metabolism. Therefore, the significant influence of MCU on the synthesis and metabolism of biomolecules such as lipids, amino acids, and nucleotides opens up a new research direction for the development of GC, and it may also provide new approaches for its treatment.

The five complexes of the oxidative respiratory chain play a crucial role in effectively coupling mitochondrial respiration and energy production. Our research has uncovered a negative correlation between MCU expression and nicotinate and nicotinamide metabolism. So high expression of MCU in gastric cancer reduces NADH. Deficiency in complex I (NADH) is the most common cause of oxidative phosphorylation disorders, which manifest with various clinical symptoms (21). Studies have reported that impaired OXPHOS function is a result of damage to complex I, leading to a shift in cellular energy metabolism towards glycolysis to sustain cell growth and ATP production (22, 23). Dysfunction of complex I also leads to increased levels of α-ketoglutarate, alterations in Hypoxia-inducible factor-1α (HIF1α), generation of ROS, and activation of pyruvate dehydrogenase kinase 2 (PDK2), which accelerates tumor growth (24). In this study, we also found a positive correlation between the MT-CO1, MT-CO2, MT-CO3, and MT-CYB with MCU. MT-CO1, MT-CO2, and MT-CO3, along with cytochrome a and cytochrome a3, form complex IV, while the proteins encoded by MT-CYB, CYC1, and UQCRFS1 constitute the electron transfer center of complex III. High expression of MCU inhibits the flow of electrons through complex I, leading to an increased transfer of electrons from complex II. When a pair of electrons from complex I is transferred to oxygen, it produces 2.5 equivalents of ATP, whereas two electrons from complex II only support the production of 1.5 equivalents of ATP. Additionally, several subunits of complex I form four proton channels on the inner mitochondrial membrane. These channels release electrons to FMN, reducing it to FMNH2 (25), which causes a conformational change in the proton channel, ultimately completing proton translocation. However, the energy change in complex II is smaller and cannot facilitate proton translocation. As a result, high expression of MCU weakens electron transfer in the oxidative respiratory chain, reduces ATP generation, and leads to mitochondrial oxidative phosphorylation dysfunction and decreased membrane potential.

Major histocompatibility complex (MHC) class I molecules play an important role in cellular immunity against pathogenic infections and cancer by delivering intracellular-derived peptides to CD8 cytotoxic T cells. MHC class II molecules play a central role in the initiation of the immune response, enabling the presentation of extracellular antigenic peptides derived from treatment to CD4 helper T cells. The expression of MHC class II and I genes is tightly regulated in the immune system (26, 27). Through analysis, we found that the high expression of MCU in gastric cancer affects the expression of MHC molecules, which in turn may reduce immature B and T Tregs while increasing memory resting CD4^+^ T cells, activated dendritic cells, and neutrophils. In addition, the expression of MCU is also related to the antigen-antibody binding reaction, which may have an important impact on the change of tumor immune microenvironment. The electron transfer in the oxidative respiratory chain also affects the immune microenvironment of GC. Regulatory T cells (Tregs) are a subset of CD4^+^ T cells that exhibit heightened mitochondrial metabolism compared to other immune cells. Research by Chandel et al. has demonstrated that the activity of complex III in the oxidative respiratory chain is essential for Treg cells to carry out their immune function (28). Recent studies have also found that mitochondria play a crucial role in the immune response of cancer patients (3). During the maturation process of T cells, immature T cells rely on OXPHOS for energy production, while mature T cells depend on glycolysis. The metabolic shift of gastric cancer cells from OXPHOS to glycolysis supports the proliferation and maturation of T cells (29, 30). Consequently, the influence of high MCU expression on the oxidative respiratory chain and OXPHOS could result in alterations in the immune microenvironment of GC tissue, thereby facilitating the proliferation of cancerous tissue.

The mitochondrial electron transport chain (ETC) is the main source of electrons needed for ROS production. Impairment of mitochondrial function inhibits ETC activity, leading to electron leakage from the ETC and promoting the production of ROS (31, 32). In normal cells, ROS are dismutated to hydrogen peroxide (H2O2) by manganese-dependent superoxide dismutase and then cleared by glutathione peroxidase. The balance between the generation and clearance of ROS maintains low levels of ROS in normal cells (33, 34). In tumor cells, the production of ROS increases, and tumor cells enhance their antioxidant systems to maintain redox balance and ensure cell survival. ROS plays a significant role in the occurrence and development of tumors (35), and the balance between ROS accumulation and antioxidant systems is involved in the cell cycle process. Consistent with numerous contemporary research findings, our study has identified a positive correlation between MCU expression and ROS. The buildup of intracellular ROS usually causes the depolarization of the mitochondrial membrane potential. This leads to the release of cytochrome C, activation of the caspase cascade, and the nuclear translocation of apoptosis-inducing factor (AIF) and endonuclease G (Endo-G), which are the primary mechanisms of mitochondria-mediated apoptosis. The accumulation of rROS and depolarization of the mitochondrial membrane potential may lead to mitochondrial swelling and increased permeability of the mitochondrial membrane, thereby triggering intrinsic apoptosis (17). This is consistent with the previously mentioned impact of MCU on the mitochondrial oxidative respiratory chain. Currently, reactive oxygen species have become a target for many anticancer drugs. Platinum-based drugs, such as cisplatin, carboplatin, and oxaliplatin, can induce tumor cell death by maintaining very high levels of reactive oxygen species (36, 37). In this study, we identified a correlation between MCU expression and resistance to platinum-based drugs, which may be attributed to the regulation of OXPHOS and ROS. In colon cancer research, it has been found that enhancing OXPHOS to generate an adequate amount of ATP can promote drug resistance by expelling drugs through multidrug transporters (38). Therefore, the MCU may be a key factor in addressing resistance to platinum-based drugs in cancer patients.

Mitochondria possess independent DNA, known as mitochondrial DNA (mtDNA), which encodes ribosomal RNA (rRNA), transfer RNA (tRNA), and essential proteins for electron transport and oxidative phosphorylation. Additionally, mtDNA has its own genetic repair mechanism (39, 40). mtDNA is essential for maintaining normal cellular function, and alterations in mtDNA are implicated in various cellular diseases. Reduction in mtDNA copy number or mutations in mtDNA indicate a decline in mitochondrial respiratory function (41, 42). Our analysis of MCU expression enrichment revealed that alterations in MCU expression can affect the biosynthesis of mtDNA and tRNA. Studies have indicated a decrease in mtDNA copy number in various cancers, including hepatocellular carcinoma (HCC), GC, and BC (43). From the gastric mucosa of healthy individuals to that of individuals in a diseased state, and further to gastric adenocarcinoma in cancer patients, there is a gradual decrease in mtDNA copy number. This observation suggests that the decrease in mtDNA copy number may be linked to the development of GC (44). The transcription initiation process in mitochondria requires mitochondrial transcription factor A (TFAM) (40). TFAM is a nuclear-encoded protein that is imported into the mitochondria and plays a crucial role in mitochondrial DNA transcription and replication. It contains two high-mobility group (HMG) box domains. Similar to other HMG family proteins, TFAM can bind, unwind, and bend mtDNA in a non-sequence-specific manner to enhance mtDNA transcription (45). Degradation of TFAM leads to mitochondrial respiratory dysfunction, increased aerobic glycolysis, redox changes, and activation of nicotinamide adenine dinucleotide phosphate (NADPH) oxidase (NOX) (46). In this study, we found a positive correlation between MCU expression and TFAM, indicating that MCU expression also influences mitochondrial DNA transcription and replication, thereby affecting mitochondrial function and metabolism.

In the end, we identified the 10 major hub genes of MCU as being MICU1, MICU2, MICU3, MCUR1, MCUB, ITPR1, ITPR3, VDAC1, SMDT1, and HSPA9. This was done by analyzing the genes and proteins that interact with MCU. These comprise the MCU complex’s MICU1, MICU2, MICU3, MCUR1, MCUB, and SMDT1. The transmembrane channel for Ca^2+^ transport is formed by MCUB and MCU. When the intracellular Ca^2+^ level rises above 3µM, MICU1, MICU2, and MICU3 function as positive regulators of MCU, physically opening the Ca^2+^ channel and causing MCU to change from a closed state to an open state. In order to stop the uptake of Ca^2+^, MCUR1 is in charge of changing MCU from an open state to a closed state. MICU1 and MICU2 are connected to MCU via SMDT1 (47). Ion channels linked to the absorption of calcium in the mitochondria are ITPR1, SMDT1, and VDAC1. Together, ITPR1 and ITPR3 regulate the cytoplasmic Ca^2+^ level via mediating the release of calcium from the endoplasmic reticulum and the release of intracellular calcium as a second messenger. On the outer membrane of the mitochondria, the voltage-dependent anion channel protein known as VDAC1 controls the flow of anions, cations, ATP, and other metabolites into and out of the mitochondria. It may work in concert with MCU, which is situated on the inner mitochondrial membrane, to control cellular metabolism, preserve intracellular calcium homeostasis, and control processes like necrosis and apoptosis. This suggests that more research is necessary. A member of the heat shock protein 70 gene family, encoded by HSPA9, is mostly found in the mitochondria and is involved in apoptosis, oxidative stress response, cell division, and mitochondrial homeostasis (48, 49). Its elevated expression in a variety of malignancies has been discovered by current study (50–52). HIF1α is regulated by it via the MAPK-ERK pathway. HIF-1α has the ability to attach to hexokinase II and VDAC1, giving cancer cells resistance to apoptosis (53). The findings of our study suggest that HSPA9 could be a downstream regulator of MCU, potentially enhancing cell division and resistance to apoptosis via triggering the MAPK-ERK pathway and HIF-1α.

The incidence, growth, metastasis, and chemoresistance of tumor cells are all significantly influenced by MCU. Although there is growing evidence that mitochondrial dysfunction is a major pathogenic factor in GC, further research is still needed to understand the specific processes. When it comes to treatment, targeting mitochondrial metabolism seems like a good idea for GC. Our current study has explored the role of MCU in GC in a preliminary manner, and the results indicate that MCU can affect mitochondrial function, energy generation, and biological metabolism, which in turn can affect the occurrence and progression of GC. Further understanding of the effects of MCU on mitochondrial function and metabolism in GC has been gained by this study, which may pave the way for future investigations into and therapies for gastric cancer.

## Data availability statement

Transcriptome data of 32 paracancer cases and 375 gastric cancer cases presented in the study are deposited in the TCGA-STAD repository, this data can be found here: https://xenabrowser.net/datapages/; Tissue data of 174 normal gastric tissuespresented in the study are deposited in the GTEx repository, this data can be found here: https://xenabrowser.net/datapages/; 45 gastric cancer and normal tissue data presented in the study are deposited in the GEO repository, accession number: GSE63089, this data can be found here: https://www.ncbi.nlm.nih.gov/geo/query/acc.cgi?acc=GSE63089. Other original contributions presented in the study are included in the article/Supplementary Material. Further inquiries can be directed to the corresponding author.

## Ethics statement

The studies involving humans were approved by Medical Ethics Committee of the Second Hospital of Lanzhou University. The studies were conducted in accordance with the local legislation and institutional requirements. The human samples used in this study were acquired from primarily isolated as part of your previous study for which ethical approval was obtained. Written informed consent for participation was not required from the participants or the participants’ legal guardians/next of kin in accordance with the national legislation and institutional requirements. Written informed consent was obtained from the individual(s) for the publication of any potentially identifiable images or data included in this article.

## Author contributions

ZX: Writing – original draft, Visualization, Methodology, Investigation, Formal analysis, Data curation, Conceptualization. XC: Writing – original draft, Validation, Software, Methodology, Investigation, Formal analysis, Conceptualization. HZ: Writing – review & editing, Validation, Supervision, Resources, Funding acquisition. LS: Writing – review & editing, Validation, Supervision, Resources. RB: Writing – review & editing, Validation, Supervision, Investigation. WY: Writing – review & editing, Validation, Supervision. JY: Writing – review & editing, Validation, Supervision. HL: Writing – review & editing, Validation, Supervision, Resources, Project administration, Funding acquisition, Conceptualization.
